# Traditional Chinese medicine intervention for autism spectrum disorders: A protocol for systematic review and network meta-analysis: Retraction

**DOI:** 10.1097/MD.0000000000029825

**Published:** 2022-09-30

The article “Traditional Chinese medicine intervention for autism spectrum disorders: A protocol for systematic review and network meta-analysis”, which appeared in Volume 101, Issue 9 of *Medicine*, is being retracted at the authors’ request due to concerns about potential overlap with other publications.
